# Degenerative Cervical Myelopathy Diagnosis and Its Differentiation from Neurological Mimics, MS and ALS: A Literature Review

**DOI:** 10.3390/jcm14248711

**Published:** 2025-12-09

**Authors:** Sydney Klumb, Lauren Haley, Chase Hathaway, Jonathan Irby, Johnny Cheng, Jacob Rumley

**Affiliations:** 1 College of Osteopathic Medicine, Rocky Vista University, Englewood, CO 80112, USA; 2 Center for Spine and Orthopedics, Thornton, CO 80229, USA

**Keywords:** degenerative cervical myelopathy, diagnostic criteria, multiple sclerosis, misdiagnosis, amyotrophic lateral sclerosis, differential diagnosis, neurological mimics, diagnostic delay

## Abstract

Multiple sclerosis (MS), amyotrophic lateral sclerosis (ALS), and degenerative cervical myelopathy (DCM) share features that may confound diagnosis. DCM is caused by degenerative changes in the cervical spine leading to spinal cord compression and injury, resulting in significant disability. Misdiagnosis of DCM for a similar neurological condition can lead to further spinal cord damage from delayed surgical treatment. Here we review the diagnostic criteria, clinical signs and symptoms, and imaging typical for DCM, and two of its clinical mimics, MS and ALS. Shared motor symptoms of all three conditions can make diagnosis difficult, especially early in disease course. Noteworthy differences include neck and shoulder pain in DCM, visual disturbances in MS, and bulbar symptoms and the absence of sensory deficits in ALS. In DCM and MS, MRI is used to support the diagnosis, with specific findings on MRI that differentiate DCM versus MS. In ALS, MRI is used to rule out differential diagnoses. Applying the diagnostic criteria for MS and ALS, as well as understanding the typical presentation and MRI findings of DCM, is crucial. Through discussion of these conditions, this review aims to help limit misdiagnosis rates, allowing for early management, which can improve long-term patient outcomes.

## 1. Introduction

Degenerative cervical myelopathy (DCM) is caused by degenerative changes in the cervical spine leading to spinal cord compression and injury, resulting in significant neurological disability [1]. DCM is estimated to affect 1 in 50 adults, or a prevalence of 2%. It is notably the most common spinal cord disorder in adults [2,3]. Established in 2019, the AO Spine REsearch Objectives and Common Data Elements for Degenerative Cervical Myelopathy (AO Spine RECODE-DCM) is an international initiative aimed at improving patient outcomes through ten research priorities [1]. Research priority 3 works toward establishing diagnostic criteria, for which there are none at present [1]. The reporting of key features of DCM in primary care is particularly low, emphasizing the need for clear diagnostic criteria [4]. Patients experience an average delay in diagnosis of 2–6 years, which is associated with further disability over this extended time period [5,6]. Early and accurate diagnosis is critical in DCM for preventing long-term disability, which significantly affects the patient’s ability to work and maintain independence [6]. Depending on severity, treatments of DCM vary, but may include close observation, or surgical approaches, either anterior, posterior, or in select cases a combined 360-degree approach. Anterior approaches include anterior cervical discectomy and fusion (ACDF), anterior cervical corpectomy and fusion (ACCF), and anterior cervical hybrid discectomy and fusion (ACHDF). Posterior approaches include laminectomy, with optional fusion and laminoplasty [7,8]. Thus, early spine surgeon referral is important in preventing or limiting long-term disability [6,9]. Delayed diagnosis can result in irreversible spinal cord damage, progression of symptoms, and worse surgical outcomes [10].

Misdiagnosis rates of DCM are currently unknown but are estimated to be significant [11]. It has been observed by the senior author that patients may be misdiagnosed with neurological conditions first. Primary differential diagnoses for DCM include, but are not limited to, multiple sclerosis, amyotrophic lateral sclerosis, syringomyelia, and spinal cord tumors [12,13]. Of many differential diagnoses, this review focused on two: multiple sclerosis (MS) and amyotrophic lateral sclerosis (ALS), as clinical mimics of DCM. MS is an autoimmune demyelinating disease of the central nervous system. Zürrer et al. in 2024 identified rates of MS misdiagnosis ranging from 5 to 41% [14]. Diagnostic delay in MS can also be significant [15]. ALS is a progressive degenerating disease of both upper and lower motor neurons. Richards et al. in 2021 noted that ALS was misdiagnosed in 13–68% of cases [16]. MS and ALS management focuses on slowing progression and improving quality of life. In MS, this is carried out through disease-modifying therapies. Patients misdiagnosed with MS receive an average of nearly 3 years of unnecessary disease-modifying therapies, leading to unresolved symptoms, adverse effects, and financial burden [17]. In ALS, patients undergo the emotional burden of receiving a prognosis with an average survival of 2–4 years, and often enroll in clinical trials to slow quality of life decline [18]. There are a few FDA-approved drugs for ALS that can extend life by 2–3 months [19]. Because management of these three conditions varies greatly, it is important for patients to be properly diagnosed initially.

Here we reviewed the diagnostic criteria, clinical signs and symptoms, and imaging typical for DCM, and two of its clinical mimics, MS and ALS. Through discussion of these conditions, this review aims to help limit misdiagnosis rates, allowing for earlier management, which can improve long-term patient outcomes.

## 2. Search Strategy

For this narrative review, a search of the database PubMed was performed. Studies in English and published in the last five years until 12 September 2024 were originally included, with an updated search up to 9 October 2025 prior to publication. The following terms were searched: cervical myelopathy AND (misdiagnosis OR “diagnostic error”), “multiple sclerosis” AND (misdiagnosis OR “diagnostic error”), “amyotrophic lateral sclerosis” AND (misdiagnosis OR “diagnostic error”), cervical myelopathy AND “diagnostic criteria”, “multiple sclerosis” AND “diagnostic criteria”, and “amyotrophic lateral sclerosis” AND “diagnostic criteria”. Search results were title- and abstract-screened for relevancy to the research question, excluding duplicates, pediatric studies, and articles not directly discussing DCM, MS, or ALS. Articles were also excluded if a full-text copy could not be obtained or if the content was not directly relevant to diagnostic differentiation. This paper included the most relevant and recent literature discussing clinical presentation, diagnostic features, and misdiagnosis patterns. Original sources cited in review articles were also reviewed and included in this paper as appropriate.

## 3. Degenerative Cervical Myelopathy

### 3.1. Diagnosis of Degenerative Cervical Myelopathy

**Figure 1:**
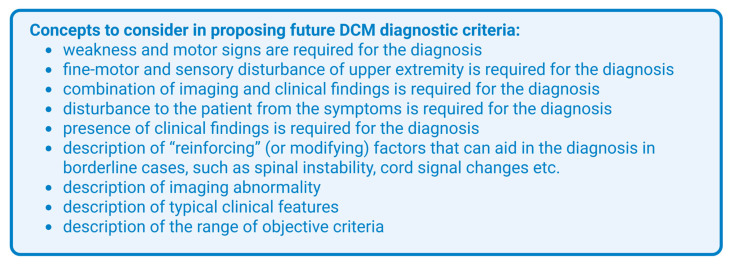
Concepts to consider in proposing future DCM diagnostic criteria, adapted from Matsoukas et al. [20].

To mitigate misdiagnoses and improve patient outcomes, there is a need to establish diagnostic criteria for DCM [1]. Adapted and shown in Figure 1, a scoping review by Matsoukas et al. in 2024 outlines concepts that should be considered in future diagnostic criteria [20]. The following data from the literature on commonly observed signs and symptoms, as well as magnetic resonance imaging (MRI) findings agreed with the outlined concepts. Further clinical research is required in developing and establishing diagnostic criteria for DCM. The DOWN questionnaire is proposed as a screening tool for DCM. The questionnaire asks patients: “1. Have you noticed that you are Dropping things or that your hands feel clumsy? 2. Have you felt more Off-balance or unsteady on your feet? 3. Do you feel Weakness in one or both of your arms or hands? 4. Do you feel Numbness or tingling in one or both of your arms or hands?” A sensitivity of 91% was found in patients who answer yes to three or more of the questions [21]. A systematic review of screening tools for primary care physicians identified both the DOWN questionnaire and the Japanese Orthopedic Association Cervical Myelopathy Evaluation (JAOCME)-derived questionnaire as the most suitable, with similar Youden indices. In this review, the authors mention that the JAOCME questionnaire does not include questions on gait imbalance and hand dysfunction, whereas the DOWN questionnaire does, and that these are important symptoms of DCM to ask about [22]. Additionally, severity of spinal cord compression symptoms in DCM are evaluated with the Nurick and modified Japanese Orthopedic Association (mJOA) scales, most commonly [6,23]. Other scoring systems include the Cooper myelopathy scale, the Prolo score, and the European myelopathy score [24].

### 3.2. Clinical Characteristics of Degenerative Cervical Myelopathy

The average age of patients with DCM is 56 years old [6]. In general, DCM presents with sensory and motor defects, and autonomic dysfunction. Patients diagnosed with DCM most commonly report symptoms of poor balance, clumsiness, neck stiffness, reduced grip strength, and hand numbness [25]. A scoping review by Jiang et al. found that the most frequent and sensitive symptoms of DCM were unspecified paresthesias (86%), hand numbness (82%), and hand paresthesias (79%). Gait impairment was seen in 72% of patients with DCM, and 51% of patients had neck and/or shoulder pain. Minorities of patients had back pain (19%), lower extremity pain (10%), bladder dysfunction (38%), bowel dysfunction (23%), and sexual impairment (4%) [26]. Lower extremity symptoms tend to direct the clinician to diagnostically work-up the lumbar spine, but these symptoms should warrant further investigation into the cervical spine too [27,28,29].

Signs and symptoms must be utilized together to diagnose DCM. A systematic review and meta-analysis of signs found that the most sensitive clinical tests were the Tromner sign and hyperreflexia, and the most specific tests were Babinski, the Tromner sign, clonus, and the inverted supinator sign. The Tromner sign showed greater sensitivity (94%) and specificity (93%) than the Hoffman sign [30]. Ultimately, upper motor neuron signs, particularly of the upper extremity, should encourage a clinician to be suspicious of DCM. These above-described clinical signs and symptoms of spinal cord compression should point clinicians towards asking the patient to complete a questionnaire, as discussed above in Section 3.1, and performing further diagnostic imaging. A flowchart approach is proposed toward diagnosis of DCM by Tetreault et al. It emphasizes the importance of MRI in diagnosis. The approach states that if upper and lower motor neuron signs or upper motor neuron signs only are present, then cervical spinal cord imaging should be obtained [31].

### 3.3. Radiographic Features of Degenerative Cervical Myelopathy

**Figure 2:**
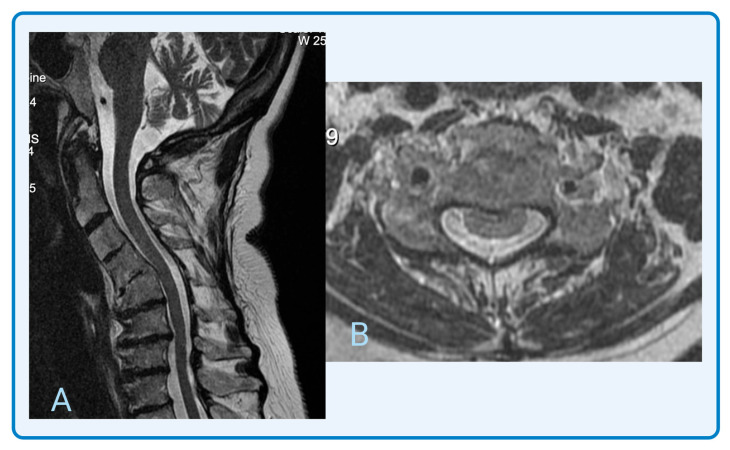
MRI cervical spine in sagittal (**A**) and transverse (**B**) views of a 65-year-old female diagnosed with DCM from the senior author’s clinical practice. This patient underwent surgical correction, resulting in severity improvement from Nurick grade IV to grade II.

Radiographs are pertinent to determine osseous pathology for DCM, as well as planning surgical intervention. T2-weighted and contrast-enhanced T1-weighted MRI is the gold standard, with a sensitivity as high as 100% [7]. MRI showing cervical spinal cord compression is key to diagnosis. Other changes may include cervical spinal canal stenosis and intramedullary T2-weighted hyperintensity signal changes in the spinal cord [13]. However, signal changes are not present in all patients with DCM, which is a limitation [13]. Using axial MRI, the cord compression ratio is calculated as the ratio between anterior–posterior diameter and transverse diameter, demonstrating deformation of the spinal cord [32]. A compression ratio <0.4 is associated with a worse prognosis [33]. Recently, a retrospective study proposed utilizing dynamic MRI techniques to capture the patient’s spinal cord in flexion and extension, as well as traditionally used neutral positioning [34]. Clinical correlation with imaging is important, because evidence does not support surgical intervention of incidental spinal cord compression on MRI in individuals who are asymptomatic [9]. Lastly, Figure 2 provides an example of a cervical spine MRI of a 65-year-old female diagnosed with DCM from the senior author’s clinical practice. This patient underwent surgical correction, resulting in severity improvement from Nurick grade IV to grade II. A narrative review by Balmaceno-Criss et al. describes DCM in great detail, including further epidemiology, risk factors, and management [13].

## 4. Multiple Sclerosis

### 4.1. Diagnosis and Radiographic Features of Multiple Sclerosis

MS is a chronic, demyelinating neurological condition with a variable disease course. The 2024 McDonald diagnostic criteria were recently agreed upon. Because this was a recent update, much of the literature reflected the 2017 McDonald criteria. The 2017 McDonald criteria diagnosed MS based on meeting both dissemination in time and dissemination in space. Dissemination in space was met by one or more T2-hyperintense lesions present in two or more central nervous system anatomic areas (periventricular, cortical, juxtacortical, and infratentorial brain regions, and the spinal cord). Dissemination in time was met by simultaneous presence of gadolinium-enhancing and non-enhancing lesions, or a new T2-hyperintense lesion on follow-up MRI compared to baseline MRI, or positive oligoclonal bands in cerebrospinal fluid (CSF) [35]. Compared to the older 2010 McDonald, the 2017 McDonald had greater specificity but lower sensitivity [36,37]. However, the 2017 McDonald criteria provided MS diagnoses significantly earlier than the previous 2010 criteria [38,39]. This is beneficial for patients, who can start on disease-modifying therapies sooner. Although, there is some concern that diagnosing patients increasingly sooner can result in greater misdiagnosis [40]. A study suggests that misapplication of the McDonald criteria is a reason for misdiagnosis, and that further education on the criteria can help with this [41]. This includes misinterpretation of abnormalities on MRI [42].

**Figure 3:**
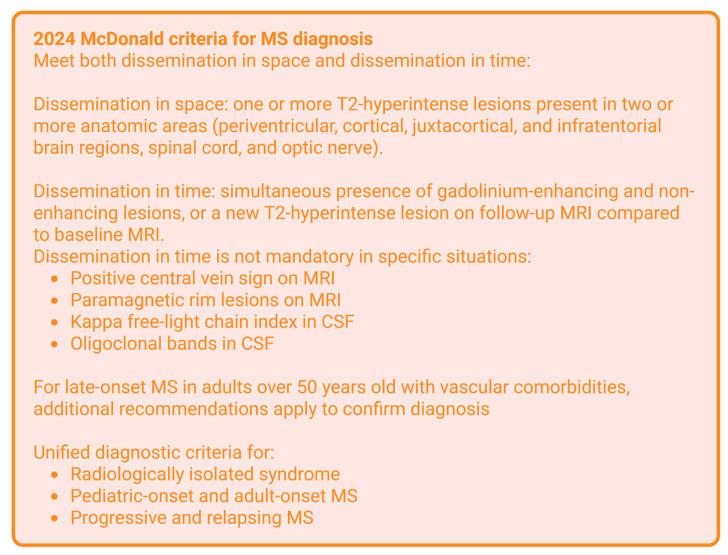
The revised 2024 McDonald criteria for MS diagnosis, adapted from Montalban et al. [43].

Figure 3 outlines a summary of the updated 2024 criteria, adapted from Montalban et al. [43]. The 2024 McDonald criteria now include the optic nerve as a fifth central nervous system area for dissemination in space [44,45,46,47]. Dissemination in time still applies but is no longer a requirement for diagnosis [48]. If a patient has lesions in at least four anatomic areas, this is sufficient to diagnose MS. Additional changes to the 2024 McDonald criteria include the central vein sign, which is when a demyelinating lesion surrounds a vein [39,49,50,51]. The select 6 rating method is used to assess for a positive central vein sign, meaning that at least 6 lesions surround a vein, or if there are less than 10 lesions, then the majority surround a vein. Thirdly, paramagnetic rim lesions, or activated iron-laden microglia around an inactive core, can be used to diagnose MS when dissemination in space is met [52,53]. Fourthly, radiologically isolated syndrome is recognized in the updated diagnostic criteria. With this, patients who have MS lesions that are incidentally found may go on to develop clinical symptoms of MS and can be diagnosed prior to symptom onset with the discussed criteria. Fifthly, patients with progressive and relapsing MS are now diagnosed under the same criteria, and the same criteria should be applied to pediatric-onset disease as well. These revisions aim to maintain the sensitivity and specificity of the 2017 McDonald and decrease time to diagnosis for patients [43].

MRI has been key for the diagnosis of MS since 2001 [39]. The 2017 criteria and Magnetic Resonance Imaging in MS (MAGNIMS) recommend brain and whole-spine MRI on all patients with a suspicious clinical presentation for MS [54]. The 2024 criteria also recommends adding an optic nerve MRI if the patient presents with optic neuritis symptoms [55]. The optic nerves can be seen on brain MRI, but they are best viewed with optic nerve MRI. Optical coherence tomography and visual evoked potential can also be used to diagnose MS under the 2024 criteria [56]. MRI findings continue to be a crucial piece of the 2024 McDonald criteria. MS is typically demonstrated on MRI as focal inflammatory demyelinating white matter lesions evident as focal hyperintensity on T2-weighted and T2-FLAIR imaging. Ideally, this is seen in at least two sequences and in at least two planes [57]. Identifying the central vein sign and paramagnetic rim lesions is limited in facilities with lower magnetic strength MRI machines [43,58]. Overall, diligently applying these criteria in Figure 3 to diagnose MS is very important for differentiating this condition from the MRI findings of patients with DCM.

CSF analysis can help to rule out other inflammatory processes of the central nervous system and is diagnostically indicated. CSF oligoclonal bands are present in 95% of patients with MS. Established in 2017, the presence of oligoclonal bands could be used to meet dissemination in time in the diagnostic criteria, in the absence of other CSF findings atypical for MS [59]. In the 2024 criteria, kappa free-light chain index in CSF can also be used interchangeably with oligoclonal bands to meet dissemination in time for diagnosis of MS [43,60,61]. Thus, positive CSF is used to diagnose patients with MRI findings that satisfy dissemination in space. If oligoclonal bands or kappa free-light chain index are present without CSF characteristics of other inflammatory conditions, MS may be the appropriate diagnosis.

### 4.2. Clinical Characteristics of Multiple Sclerosis

Typical clinical signs and symptoms of MS are unilateral painful vision changes, motor weakness, sensory deficits, and brainstem and cerebellar dysfunctions, including imbalance, incoordination, diplopia, or vertigo [40,54]. Symptoms usually last a minimum of 24 h but can persist. Patients may also experience fatigue, mood changes, and cognitive dysfunction [40]. Signs include reduced fine touch, vibration sense and joint position sense, increased tone, hyperreflexia, and upward-going Babinski. Eye exam may demonstrate isolated sixth nerve palsy and/or internuclear ophthalmoplegia. If bilateral internuclear ophthalmoplegia is present, this is pathognomonic of MS [62]. An MS lesion in the cervical spinal cord can cause Lhermitte’s sign, which is the sensation of an electric shock down the neck when flexing the neck [62]. This is a sign also present in DCM [7].

### 4.3. Late-Onset Multiple Sclerosis

MS most commonly develops in patients between 20 and 40 years old. Late-Onset MS (LOMS), diagnosed after age 50, makes up about 5% of MS cases [63]. LOMS can complicate the differential, given that MS usually has a younger onset. This is another opportunity for misdiagnosis, especially in discussion with DCM. The first presentation of LOMS is usually motor dysfunction [63]. In patients older than 45, the accuracy of the MS diagnosis can be improved by requiring at least three periventricular lesions on MRI, given that these lesions can appear as an age-related phenomenon in patients with cardiovascular risk factors [39]. Steinegger et al. also found that patients with LOMS were less likely to present with gadolinium-enhancing lesions on MRI, which likely contributes to further diagnostic challenges in this age group [64]. Considering these limitations, the updated 2024 McDonald criteria recommends that diagnosis of older patients with MS should require features such as a spinal cord lesion, positive central vein sign, or CSF with positive oligoclonal bands or kappa free-light chain index [43]. The updated 2024 criteria are found to have preserved sensitivity for diagnosing LOMS compared to previous criteria [65].

## 5. Amyotrophic Lateral Sclerosis

### 5.1. Diagnosis of Amyotrophic Lateral Sclerosis

**Figure 4:**
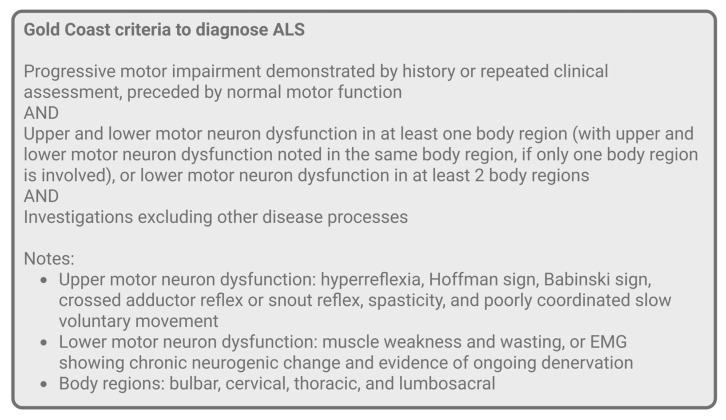
The new 2020 Gold Coast criteria to diagnose ALS, adapted from Shefner et al. [67].

ALS is a progressive degenerative disease of motor neurons. Past diagnostic criteria for ALS were the El Escorial and the Awaji. The El Escorial criteria were developed for research purposes but inevitably used for clinical practice, and the opposite was true for the Awaji criteria. In 2020, the Gold Coast criteria were developed for both research and clinical practice diagnosis of ALS [19,66]. Adapted from Shefner et al., Figure 4 outlines the new criteria [67]. According to Pugdahl et al., the new Gold Coast criteria have demonstrated an increased sensitivity at 88% and a preserved high specificity compared to the El Escorial and the Awaji [68]. A study by Ferullo et al. also found an increased sensitivity of the Gold Coast criteria compared to the previous two criteria, but a lower specificity [69]. Though improved, as argued by Ferullo et al., the current criteria still pose challenges for providing timely diagnosis to patients [70]. Additionally, there are two main staging systems for assessing the progression of ALS, including the King’s Clinical staging system and the Milano–Torino (MiToS) Functional staging system, the latter based on the Amyotrophic Lateral Sclerosis Functional Rating Scale—Revised (ALSFRS-R) [71].

### 5.2. Clinical Characteristics of Amyotrophic Lateral Sclerosis

The average age of patients with ALS is 57 years old, similarly to DCM [72]. Clinically, ALS tends to present as bulbar-dominant type or limb-dominant type. Bulbar symptoms include dysphagia, dysarthria, drooling, and chewing difficulty [72]. Fasciculations and atrophy of the tongue are signs of bulbar involvement [73]. Limb symptoms include weakness of the upper or lower extremities and a loss of hand dexterity [72]. As well as hand clumsiness, patients report foot drop. Lower motor neuron involvement can look like muscle cramps and fasciculations with associated weakness. Upper motor neuron involvement can look like hyperreflexia, spasticity, and the Hoffman or Tromner sign. The bulbar-dominant type is known for resulting in a shorter duration between onset and diagnosis, compared to the limb-dominant type [72]. This is likely due to the similarity in symptoms between limb-dominant type and other mimicking conditions, like DCM. The majority of patients with ALS have limb-onset type compared to bulbar [19]. ALS tends to progress from one body segment to the contralateral side and then to adjacent body areas. Regardless of dominant body region, ALS presents with a combination of upper motor neuron and lower motor neuron dysfunction [74]. Motor symptoms progress to proximal muscles. Eventually this affects respiratory muscles and causes dyspnea at rest [73]. Some research is beginning to discuss and characterize pain symptoms in patients with ALS [75,76]. Up to 30% of patients may experience pseudobulbar affect, which is uncontrollable spells of laughing or crying. About 50% of patients have cognitive or behavioral dysfunction [73]. Unlike ALS, bulbar or pseudobulbar symptoms would not be expected in DCM. Further differentiating the two conditions, DCM usually does not present with motor symptom progression, but does present with prominent and/or early sensory and autonomic symptoms, and bowel and bladder incontinence [18].

### 5.3. Other Considerations in Amyotrophic Lateral Sclerosis

In ALS, neuroimaging is indicated in the work-up. However, MRI is used to rule out differential diagnoses, such as MS and DCM, rather than to diagnose ALS [73]. Electromyography (EMG) can be helpful in ruling in lower motor neuron involvement, but should only play a supplemental part in the diagnosis process [77]. Patients with ALS can appear to have carpal tunnel syndrome [19].

Familial ALS only accounts for 5–10% of cases [72]. The most common gene variants for familial ALS are the SOD1 variant and C9orf72 expansion [73,78]. Genetic testing may be helpful to confirm these cases, if family history demonstrates a possible genetic connection. However, this decision should be made carefully, weighing the risks and benefits of genetic testing [73].

## 6. Discussion

Although they are three different pathologies, the patient’s presenting clinical signs and symptoms can make differentiating DCM, MS, and ALS difficult. It is important to gather a clear symptom history from the patient, along with a complete neurologic physical exam. When there is clinical uncertainty, applying the diagnostic criteria for MS and ALS, as well as understanding the typical presentation and MRI findings of DCM, is crucial. Diagnostic criteria are not yet established for DCM. This greatly impacts research on DCM and patient outcomes, as noted by the AO Spine RECODE-DCM [1]. In writing this review, it was found that several papers, including one by Sharma et al., aim to provide evidence-based approaches to diagnosis of DCM [20,31,79], but none provide diagnostic criteria yet.

**Figure 5:**
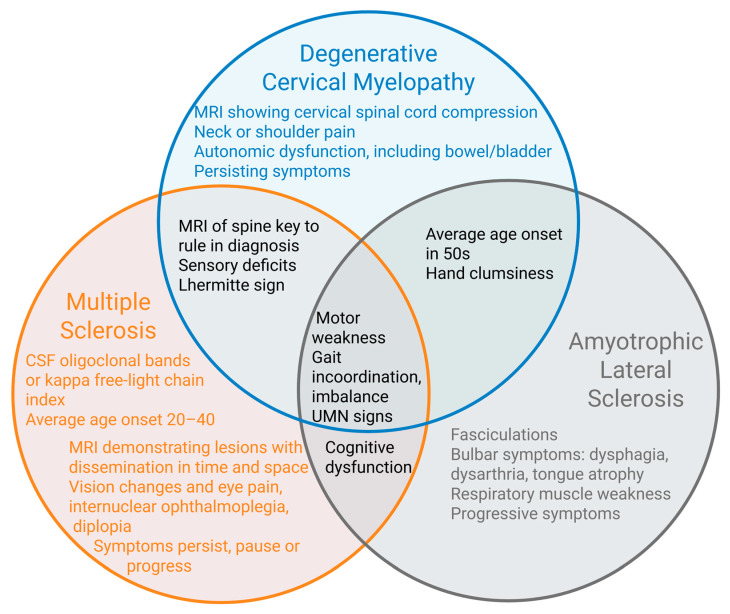
Demonstrating similarities and differences between clinical presentations of DCM, MS, and ALS.

To our knowledge, prior summaries have not compared and contrasted DCM with similar conditions in the manner presented in this review. Highlighting this, Figure 5 demonstrates and summarizes the overlapping features of each condition and their defining features. Noteworthy differences include neck and shoulder pain in DCM, visual disturbances in MS, and bulbar symptoms and the absence of sensory deficits in ALS. In DCM and MS, MRI is used to support the diagnosis and rule out differential diagnoses, with specific findings on MRI to differentiate DCM versus MS. In ALS, MRI is used to rule out differential diagnoses [2]. Shared motor symptoms of all three conditions can make diagnosis difficult, especially early in disease course. This review has described typical presentations of DCM, MS, and ALS. However, the author would like to acknowledge that atypical features exist in each condition, with which the patient may present. The goal is to prevent misdiagnosis in as many patients as possible, with the understanding that diagnostic criteria are without 100% sensitivity and specificity. The literature reflects the need for improved biomarkers and diagnostic tools to distinguish between these conditions earlier. Many are under investigation for MS and ALS, as well as DCM [80,81,82,83,84,85,86,87]. A prospective study by Vedantam et al. discusses presurgical levels of neurofilament light chain, interleukin-6, and brain-derived neurotrophic factor as potential diagnostic biomarkers for DCM [84]. Lanza et al. describe the evaluation of patients with DCM using motor-evoked potentials to transcranial magnetic stimulation [85]. These and other biomarkers and diagnostic tools for these conditions are a valuable area for future research.

Incidence of DCM is anticipated to increase with our aging population in the United States [7]. In alignment with the AO Spine RECODE-DCM, the establishment of diagnostic criteria for DCM can significantly aid future patients. Further education on current diagnostic criteria for MS and ALS and the development of diagnostic criteria for DCM are crucial for reducing misdiagnosis [1,41]. In the event that misdiagnosis does occur, Baufeldt et al. have published guidelines to reference for communicating a misdiagnosis of MS to patients [88]. Significantly more articles have been published on MS and ALS than DCM, emphasizing the need for more research on DCM. This review hopes to contribute to the AO Spine RECODE-DCM research priority to help raise awareness and understanding of DCM. In improving misdiagnosis rates, patients can obtain access to appropriate management earlier, which can improve long-term patient outcomes. If a patient cannot be definitively diagnosed with a neurological condition by its respective diagnostic criteria, then a referral to an orthopedic spine surgeon for DCM evaluation is recommended. In other cases, orthopedic spine surgeons may refer to neurologists after determining a patient does not have DCM. When DCM, MS, and ALS are on the differential list, sending the patient for brain and spine MRI is prudent. All clinicians should remain vigilant for DCM as a potential underlying pathology. The importance in understanding the clinical presentations is universal because, anecdotally, patients may experience months-long waits for referral appointments with specialists. Preventing further delays in care with the correct diagnosis via understanding and differentiating the respective clinical presentations of DCM and mimics like MS and ALS is stressed.

## 7. Conclusions

Differentiating DCM from MS and ALS remains a significant clinical challenge due to overlapping motor and sensory features. Recognizing distinguishing symptoms and MRI characteristics is essential for timely and accurate diagnosis, which directly impacts patient management and outcomes. Appropriately applying the diagnostic criteria for MS and ALS is important. Continued research into establishing diagnostic criteria for DCM and novel biomarkers for all three conditions is critical to reduce misdiagnosis and improve early identification of these conditions.

## 8. Limitations and Future Directions

This paper is a narrative review, which naturally lends itself to limitations that are not present in systematic or scoping reviews. While a thorough review of the most relevant and recent literature was conducted, it may be subject to selection bias because articles were not systematically screened according to PRISMA guidelines, and formal risk-of-bias assessment or quality appraisal was not conducted. This review is also limited to discussion of two of many differential diagnoses related to DCM.

Future papers may discuss comparison and contrast of other differential diagnoses for DCM, including but not limited to syringomyelia, spinal cord tumors, normal pressure hydrocephalus, hereditary spastic paraparesis, metabolic myelopathy, neuromyelitis optica spectrum disorder, and peripheral neuropathy, to further aid in avoiding misdiagnosis of patients and providing the appropriate treatments. Establishing diagnostic criteria for DCM is of the utmost importance. Another area for future research and review is a focus on biomarkers and other novel diagnostic tools, which this review has introduced but has not discussed in detail.

## Data Availability

The original contributions presented in this study are included in the article. Further inquiries can be directed to the corresponding author.
